# Biosynthesis of Taxadiene in *Saccharomyces cerevisiae *: Selection of Geranylgeranyl Diphosphate Synthase Directed by a Computer-Aided Docking Strategy

**DOI:** 10.1371/journal.pone.0109348

**Published:** 2014-10-08

**Authors:** Ming-zhu Ding, Hui-fang Yan, Lin-feng Li, Fang Zhai, Lu-qing Shang, Zheng Yin, Ying-jin Yuan

**Affiliations:** 1 Key Laboratory of Systems Bioengineering, Ministry of Education, & School of Chemical Engineering and Technology, Tianjin University, Tianjin, P.R. China; 2 Collaborative Innovation Center of Chemical Science and Engineering, Tianjin, P.R. China; 3 State Key Laboratory of Medicinal Chemical Biology & College of Pharmacy, Nankai University, Tianjin, P.R. China; RIKEN Advanced Science Institute, Japan

## Abstract

Identification of efficient key enzymes in biosynthesis pathway and optimization of the fitness between functional modules and chassis are important for improving the production of target compounds. In this study, the taxadiene biosynthesis pathway was firstly constructed in yeast by transforming *ts* gene and overexpressing *erg20* and *thmgr*. Then, the catalytic capabilities of six different geranylgeranyl diphosphate synthases (GGPPS), the key enzyme in mevalonic acid (MVA) pathway catalyzing famesyl diphosphate (FPP) to geranylgeranyl diphosphate (GGPP), were predicted using enzyme-substrate docking strategy. GGPPSs from *Taxus baccata x Taxus cuspidate* (GGPPSbc), *Erwinia herbicola* (GGPPSeh), and *S. cerevisiae* (GGPPSsc) which ranked 1^st^, 4^th^ and 6^th^ in docking with FPP were selected for construction. The experimental results were consistent with the computer prediction that the engineered yeast with GGPPSbc exhibited the highest production. In addition, two chassis YSG50 and W303-1A were chosen, and the titer of taxadiene reached 72.8 mg/L in chassis YSG50 with GGPPSbc. Metabolomic study revealed that the contents of tricarboxylic acid cycle (TCA) intermediates and their precursor amino acids in chassis YSG50 was lower than those in W303-1A, indicating less carbon flux was divided into TCA cycle. Furthermore, the levels of TCA intermediates in the taxadiene producing yeasts were lower than those in chassis YSG50. Thus, it may result in more carbon flux in MVA pathway in chassis YSG50, which suggested that YSG50 was more suitable for engineering the taxadiene producing yeast. These results indicated that computer-aided protein modeling directed isoenzyme selection strategy and metabolomic study could guide the rational design of terpenes biosynthetic cells.

## Introduction

In the past few years, producing natural products by synthetic biology strategies has attracted more and more attention [1]–[3]. More than 50,000 compounds belong to terpenoids or isoprenoids which are important secondary metabolites [4]. Taxadiene is one of the key precursors for the synthesis of anti-cancer drug taxol. A large number of studies have shown that inducing the functional modules into *Saccharomyces cerevisiae* or *Escherichia coli* chassis is a feasible way for terpenes production [5]–[18]. Production of taxadiene has reached a yield of 1020 mg/L in *E. coli* after fermentation optimization [18]. However, *E. coli* platform may not be suitable for downstream processing and enzyme modifying though it is capable for the initial biosynthetic steps for terpene hydrocarbons [19]. Contrarily, *S. cerevisiae* is more suitable for further construction for synthesis steps and industrial production [15], [20], [21]. One of the advantages is that yeast could provide the biosynthetic machinery necessary for modifying the downstream enzymes, such as P450 hydroxylases [20]. Moreover, yeast has the possibility to harness different subcellular compartments for the production of natural products, such as plant terpenoids [15]. Most importantly, yeast is robust that it can withstand severe conditions such as reduced pH and high osmotic pressure, and is not susceptible to phage infections [21]. Unfortunately, production of taxadiene in yeast has not been able to meet the expectation. It has only a yield of 8.7 mg/L according to our knowledge [13]. Improvement of the terpene production is of great value for synthetic biology research.

A common strategy to improve the terpene production is to increase the endogenous supply of precursor metabolites [12]–[15], [22]. The ability to enhance heterologous production of a target compound may be limited by inability of the heterologous enzymes to collaborate with the native enzymes [23]. The content of geranylgeranyl diphosphate (GGPP) in *S. cerevisiae* [24] is very low that improving the GGPP content is necessary for further improvement of the terpene production in yeast. Geranylgeranyl diphosphate synthase (GGPPS) exists in a variety of plants, animals and microbes with the similar functions of catalyzing DMAPP or FPP to GGPP. However, the catalytic capabilities of GGPPSs may be different. Thus, the selection of GGPPS is very important for their catalytic capabilities and fitness with yeast chassis. The catalytic efficiency is determined by biochemical properties of the isoenzymes. The selection of isoenzymes should take consideration of the properties of each enzyme. In the case of GGPPS, only limited *in vitro* data of isoenzymes are available [25]. In lack of characterization data of isoenzymes, conventionally, the selection of isoenzymes has to reply on several rounds of random screening which is time consuming and labor intensive. A rational approach that could facilitate the selection of isoenzymes would benefit significantly the design and optimization of the biosynthesis. Computational simulation methods has been applied in previous metabolic engineering efforts to model cellular metabolism and predict gene deletion or over-expression targets to improve specified metabolite levels [26]. Structure based drug design has been widely used to predict the preferred binding orientation, the affinity and the activity of the drug candidate towards a specific protein [27], [28]. Hence, it is thought that computer aided protein modeling and docking study may provide us a feasible way to predict the binding affinity of enzymes with a specific substrate. In lack of biochemical data, the enzymes with better performance might be identified through guidance of computer aided protein modeling and docking study which would benefit for the rational design and efficient construction.

In this study, six different GGPPSs from *Taxus baccata x Taxus cuspidate* (GGPPSbc), *Ginkgo biloba* (GGPPSgb), *Rana catesbeiana* (GGPPSrc), *Erwinia herbicola* (GGPPSeh) and *Chlamydomonas reinhardtii* (GGPPScr) and *S. cerevisiae* (GGPPSsc) were used to predict its fitness with its substrate FPP by protein modeling and docking strategy, which guided the designing and constructing of a yeast strain with the taxadiene production of 72.8 mg/L. Metabolomics study is a commonly used strategy to identify the metabolic differences between different strains. Thus, the different chassis strains were analyzed by metabolomics to identify the more suitable chassis. The approaches used in this study demonstrate a new strategy for rational and efficient design in synthetic biology for taxadiene and other terpenes production in the future.

## Materials and Methods

### Strains and vectors

*S. cerevisiae* W303-1A MATa (*leu2-3,112, trp1-1, can1-100, ura3-1, ade2-1, his3-11,15*) was purchased from ATCC. *S. cerevisiae* YSG50 MATα (*ade2-1, ade3Δ22, ura3-1, his3-11,15, trp1-1, leu2-3,112, can1-100*) was obtained from the lab of Huimin Zhao in University of Illinois. *E. coli* DH5α (*endA1, hsdR17, gyrA96, thi-1, relA1, supE44, recA1, ΔlacU169* (Φ80lacΔZM15)) which was used for transformation and plasmids extraction was purchased from BEIJING Biomed Co., Ltd. The yeast strains used in this study were listed in Table 1. The cloning plasmid pUC18 (purchased from Takara Biotechnology (DALIAN) Co., Ltd.) and the yeast expression plasmids pRS304, pRS403, pRS305 and pRS425 (purchased from Addgene (American)) were used in this study.

**Table 1:** Strains used in this study.

Strain No.	Chassis	Description	Sources
	W303-1A	none	Purchased
	YSG50	none	Prof. Zhao
SyBE_001103	YSG50	pRS403-*tdh3p-erg20-cyct*; pRS304-*tdh3p-thmgr-cyct*	This study
SyBE_001104	W303-1A	pRS403*-tdh3p-erg20-cyct*; pRS304*-tdh3p-thmgr-cyct*	This study
SyBE_001188	W303-1A	pRS425-*tdh3p-ts-pgkt*	This study
SyBE_001189	W303-1A	pRS425*-tdh3p-ts-pgkt-pgkp-bts1-cyct*	This study
SyBE_001190	W303-1A	pRS403*-tdh3p-erg20-cyct*; pRS425*-tdh3p-ts-pgkt-pgkp-bts1-cyct*	This study
SyBE_001109	W303-1A	pRS403*-tdh3p-erg20-cyct*; pRS304*-tdh3p-thmgr-cyct*; pRS425*-tdh3p-ts-pgkt-pgkp-ggpps-cyct*	This study
SyBE_001110	W303-1A	pRS403*-tdh3p-erg20-cyct*; pRS304*-tdh3p-thmgr-cyc*t; pRS425*-tdh3p-ts-pgkt-pgkp-bts1-cyct*	This study
SyBE_001111	W303-1A	pRS403*-tdh3p-erg20-cyct*; pRS304*-tdh3p-thmgr-cyct*; pRS425*-tdh3p-ts-pgkt-pgkp-crtE-cyct*	This study
SyBE_001113	YSG50	pRS403*-tdh3p-erg20-cyct*; pRS304*-tdh3p-thmgr-cyct*; pRS425*-tdh3p-ts-pgkt-pgkp-ggpps-cyct*	This study
SyBE_001114	YSG50	pRS403*-tdh3p-erg20-cyct*; pRS304*-tdh3p-thmgr-cyct*; pRS425*-tdh3p-ts-pgkt-pgkp-bts1-cyct*	This study
SyBE_001115	YSG50	pRS403*-tdh3p-erg20-cyct*; pRS304*-tdh3p-thmgr-cyct*; pRS425*-tdh3p-ts-pgkt-pgkp-crtE-cyct*	This study

### Construction of yeast expression vectors

**Figure 1:**
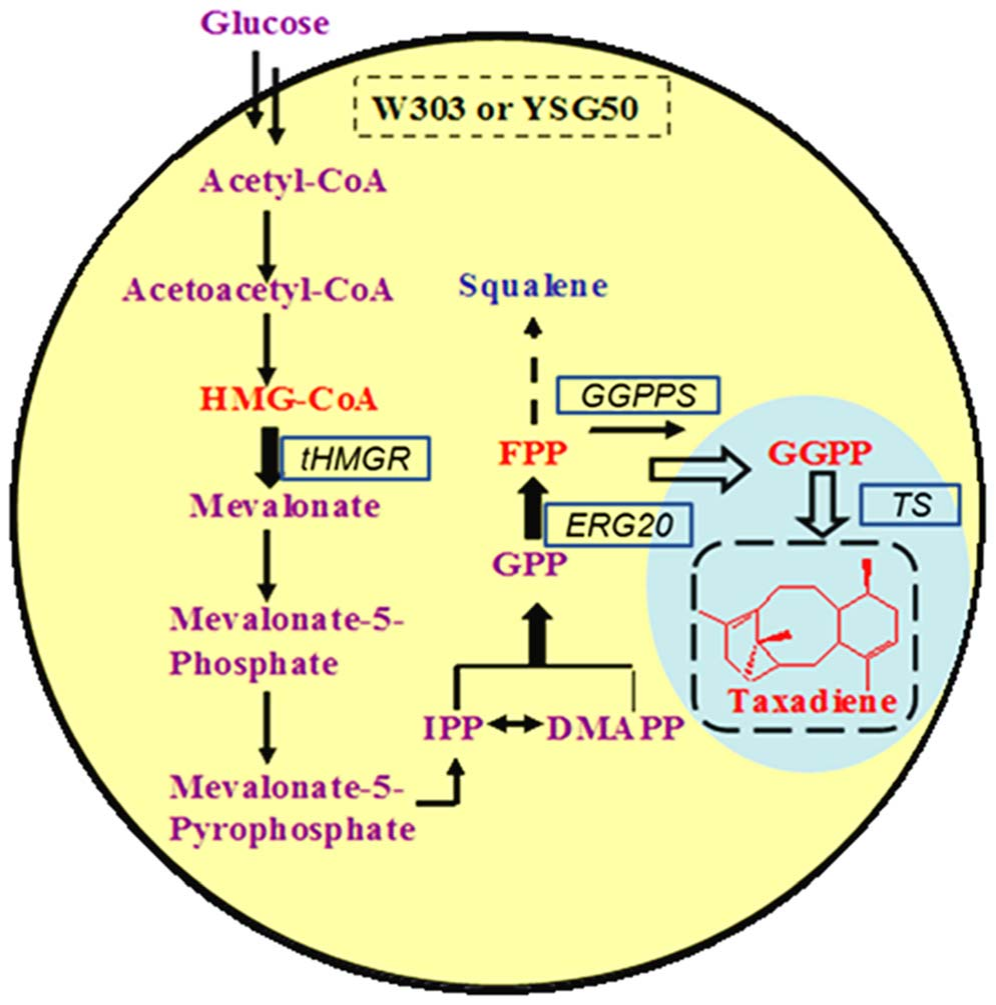
The engineered taxadiene biosynthetic pathway in *S. cerevisiae*.

The taxadiene producing yeast was designed as shown in Fig.1A. For the production of taxadiene, vectors containing *ts* from *T. brevifolia* and *ggppsbc* from *T. baccata x T. cuspidate* (or *ggppssc* from *S. cerevisiae* (*bts1*), *ggppseh* from *E. herbicola* (*crtE*)) were constructed, respectively (Fig.1B). The codon optimization of *ts, ggppsbc* and *ggppseh* were performed by AuGCT Company (China). All the primers used in this study were listed in Table S1 in File S1. The *bts1* was amplified by PCR from the genomic DNA of W303-1A using primers 13-14. To express the *ts* gene separately, the *tdh3p-ts-pgkt* cassette was constructed by OE-PCR [29] using primers 15-20. Then the 3.6 kb fragment was cleaved with *Sac I* and *Hind III*, and was introduced into the corresponding sites of vector pRS425, yielding plasmid pRS425-*tdh3p-ts-pgkt*. In order to obtain pRS425*-ts-bts1*, pRS425*-ts-crtE* and pRS425*-ts-ggppsbc*, the *pgkp-bts1-cyct, pgkp-crtE-cyct* and *pgkp-ggppsbc-cyct* cassettes were constructed by OE-PCR using primer 21–38. The fragments were all cleaved with *Hind III* and *Apa I* and introduced into the corresponding sites of plasmid pRS425-*tdh3p-ts-pgkt*, respectively. To generate an integrated plasmid pRS305*-ts-ggppsbc*, the *tdh3p-ts-pgkt-pgkp-ggppsbc-cyct* cassette was cleaved with *SacI* and *ApaI* from plasmid pRS425*-ts-ggppsbc*, and introduced into the corresponding sites of vector pRS305.

The *thmgr* and *erg20* are encoding genes of the MVA pathway in *S. cerevisiae*. The plasmids pRS304-*tdh3p-thmgr-cyct* and pRS403-*tdh3p-erg20-cyct* containing the *thmgr* and *erg20* module respectively (Fig. 1C) were constructed to improve the accumulation of FPP in yeast. To generate pRS304-*tdh3p-thmgr-cyct*, the *tdh3p*, *thmgr* and *cyct* were amplified by PCR from the genetic DNA of W303-1A using primers 1-6. Then the fragments were cleaved with *Apa I/ECoR I*, *ECoR I/Pst I* and *Pst I/BamH I*, which were then introduced into the corresponding sites of vector pRS304. Similarly, the *tdh3p*, *erg20* and *cyct* were amplified by PCR using primers 7–12. Fragments were cleaved with *Xba I/BamH I, BamH I/ECoR I* and *ECoR I/Apa I*, respectively, and introduced them into the corresponding sites of vector pRS403 to obtain vector pRS403-*tdh3p-erg20-cyct*.

### Yeast transformation, verification and cultivation

Yeast was transformed using the LiAc/SScarrier DNA/PEG method followed by selection for prototrophic growth on SD-drop agar plates (0.2% amino acid mixture, 0.67% yeast nitrogen base without amino acid, 2% glucose, 1.8% agar) without supplementation of the appropriate metabolite. After 3 days incubation at 30°C, five colonies were selected to cultivate in SD-drop medium at 220 rpm. When the OD_600_ reached about 4.0, the plasmids or genome was extracted. The colonies were verified by PCR with the plasmids or genome as template to avoid the false positive. The strains constructed in this study (Table 1) were all obtained as described above.

For taxadiene production, yeast was firstly inoculated into 20 mL culture tubes containing 5 mL SD medium and cultivated at 30°C, 200 rpm until OD_600_ reached about 4.0. Aliquots were diluted to an initial OD_600_ of 0.05 in 50 mL of SD or YPD medium in 250 mL flasks and were cultivated at 30°C, 200 rpm. Yeast cultures were harvested for analysis of taxadiene after 60 h.

### Extraction, purification and analysis of taxadiene

In order to purify the taxadiene, strain SyBE_001113 were cultured in the 5 L bioreactor with 2 L SD medium. The engineered yeast was prepared in a shake flask at 30°C, 200 rpm till the OD_600_ reached 4.0. The seed was then transferred to the bioreactor with an initial OD_600_ of 0.05, and was cultivated at 350 rpm and 30°C for 66 h with aeration of 1 vvm. The pH was controlled at 5.7. Then 300 ml n-hexane was added to the bioreactor, and stirred for 3 h. The n-hexane phase was pooled and evaporated in vacuum, and the residues were subjected to column chromatography on silica gel eluted with n-hexane for purification. Gas chromatography coupled to time-of-flight mass spectrometry (GC-TOF/MS, Waters) and NMR (Bruker) were used to further identify the purified taxadiene (Fig. S1a and S1b in File S1).

Fermentation cultures (400 µl) of the engineered *S. cerevisiae* were extracted with the same volume of n-hexane. The mixture was vortexed for 20 min, and 10 µl of the n-hexane phase was analyzed by GC-TOF/MS to quantify the taxadiene. The purified taxadiene was used as standard for qualification by external standard method.

### Extraction and derivatization of intracellular metabolites

Yeast cells were harvest during middle log phase and stationary phase. The samples were quenched and extracted as described previously [30]. Firstly, cells were washed with cold water and centrifuged at 3,500 rpm for 3 min. Then cells were quenched in liquid nitrogen for 3 min to arrest metabolism instantaneously. After that, cells were suspended with 1 ml extraction buffer of methanol/water (1∶1, v/v, −40°C) and mixed thoroughly. The mixture was frozen in liquid nitrogen (1 min) and then thawed for three times. After centrifugation, the supernatant was collected, and then 0.5 ml of extraction buffer was added to the cells. The centrifugation at 12,000 rpm for 5 min was followed and the extract was combined with the former one. Then 10 µl internal standard solution (succinic *d_4_* acid, 0.2 mg/ml) was added to 100 µl extract aliquot before lyophilization. Four biological replicates were performed for each sample.

Two-stage chemical derivatization was performed on dried metabolite samples as described previously [31]. Firstly, methoximation of the carbonyl groups was carried out by dissolving sample in 50 µl methoxamine hydrochloride (20 mg/ml in pyridine) and incubating it at 40°C for 80 min. Then, 80 µl *N*-methyl-*N*-(trimethylsilyl) trifluoroacetamide (MSTFA) was added and the sample was incubated at 40°C for 80 min for trimethylsilylation.

### GC-TOF/MS analysis of extracellular taxadiene and intracellular metabolites

One microliter sample was injected by Agilent 7683 autosampler into Agilent 6890 GC which was equipped with a fused-silica capillary column (30 m×0.25 mm i.d., 0.25 µm DB-5MS, J&W Scientific, Folsom, CA). The injector temperature was 260°C, and ions were generated by a 70 eV electron beam at an ionization current of 40 µA. The column effluent was introduced into the ion source (250°C) of TOF/MS. The mass scan range was 50–800 m/z.

For GC-TOF/MS analysis of taxadiene, the temperature gradient program started at 200°C for 3 min followed by heating the column at 4°C/min to 270°C, and a final constant hold at 270°C for 2 min. The taxadiene was identified by the mass fragment m/z 272, 122, and 107 according to the identification of purified sample (Fig. S2 in File S1). For GC-TOF/MS analysis of intracellular metabolites, the oven temperature was programmed as: 70°C for 2 min, then increased to 290°C (5°C/min), holding for 3 min.

### Protein Modeling

To embark on simulating protein structures of the six different GGPPSs, the template of each GGPPS was sought out by Basic Local Alignment Search Tool (BLAST) and selected with respect to multi-factors including total score, query coverage, identity and E-value (Table S2 in File S1). The sequences were well aligned with their corresponding templates, and homology models were subsequently built and refined in an Amber12EHT force field. All above mentioned modeling processes were exerted by the Protein module embedded in the Molecular Operating Environment (MOE) software package [32].

The molecular docking was run by the software AutoDock 4.2 [33] on a Linux workstation to predict the binding affinities of six different GGPPSs with FPP. In the process of preparing the ligand and receptors, waters and inconsequential ions were removed while charges were added. For each protein, the grid box, which delimited the space of ligand's movement, was set to rightly contain the whole binding pocket, referring to the position of ligand in corresponding template. Lamarckian Genetic Algorithm (LGA) was applied to execute the docking calculation. The output conformations of each docking were ranked, clustered and analyzed in AutoDock Tools, rendering the most convincing binding poses.

### Western blotting

Total proteins were prepared from yeast cells with an extraction buffer (2 mL/g cells) containing 50 mM 4-(2-hydroxyerhyl) piperazine-1-erhanesulfonic acid (HEPES) (pH 7.4), 10% glycerol (v/v), 10 mM EDTA, 0.1% Triton X-100 (v/v), 200 µM phenylmethyl sulfonyl fluoride (PMSF), and 2 µg/mL each of aprotinin, leupeptin, and pepstatin A.

Proteins were separated by SDS-PAGE on 10% polyacrylamide gels, and the polypeptides were transferred to PVDF membranes (0.22 µM, Amersham Life Science, Little Chalfon, UK) in a medium consisting of 25 mM Tris-HCl (pH 8.3), 192 mM glycine and 20% (v/v) methanol. After rinsing in the Tris-buffered saline (TBS) containing 10 mM Tris-HCl (pH 7.5) and 150 mM NaCl, the blotted membranes were pre-incubated for 2 h in a blocking buffer containing 5% (w/v) non-fat milk dissolved in TBS supplemented by 0.05% (v/v) Tween-20 (TBST1) and then incubated with gentle shaking for 2 h at room temperature in the appropriate antibodies (Anti-FLAG, Sigma, diluted 1∶10000 in the blocking buffer). Anti-alpha Tubulin (Abcam, ab184970, 1∶1000) was used as a loading control. Following extensive washes by TBST1, the membranes were incubated with Rabbit anti-mouse IgG conjugated with horse radish peroxidase (1∶5000 diluted in TBST1) at room temperature for 2 h and then washed with TBST2 (50 mM Tris-HCl, pH 7.5, 150 mM NaCl, 0.1% (v/v) Tween-20) and TBS. The locations of antigenic proteins were visualized by scanning the membranes with Las 500 machine (GE. USA).

### Real-time PCR analysis

Transcription of GGPPSbc, GGPPSeh, and GGPPSsc was evaluated by real-time PCR. The extraction of total RNA was performed using TriZol solution (invitrogen). Real-time PCR was performed in a total volume of 20 µL containing diluted cDNA (1 µL), 10 µL of 2×SsoFast supermix, 0.8 µL of each primer (10 µM final concentration), 0.4 µL of 50× ROX Reference Dye II. PCR was run on a CFX96 real time PCR system (Bio-Rad). The cycling conditions used were 95°C for 2 min, followed by 40 cycles of 95°C for 10 s and 58°C for 20 s. A no-template control was included on each reaction plate. Relative expression levels of the target genes were normalized to Actin. Each experiment was repeated three times.

## Results and Discussion

### Production of taxadiene in yeast by improving endogenous FPP and GGPP supply

**Figure 2:**
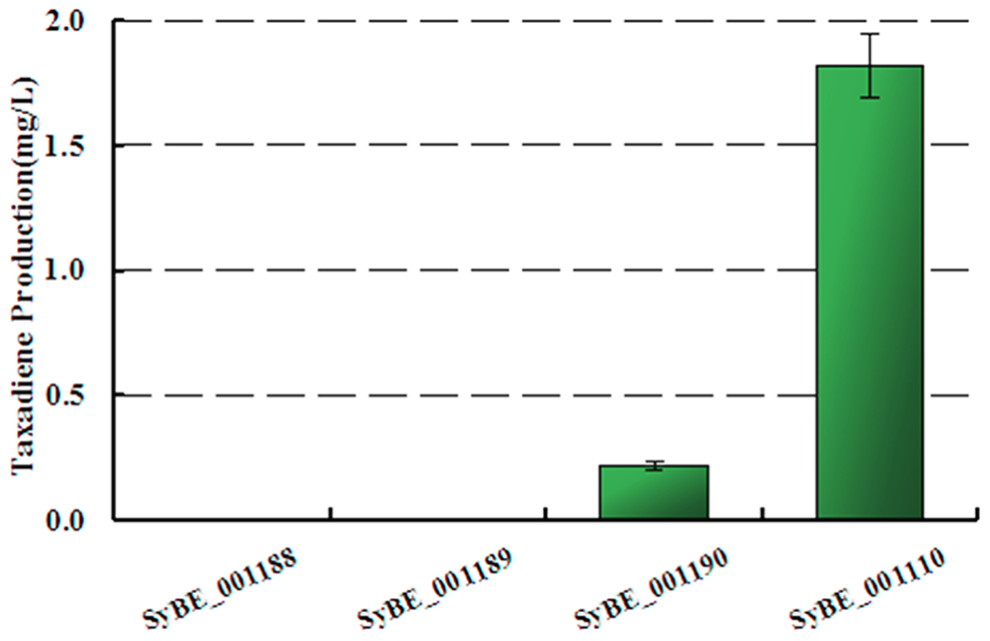
Production of taxadiene by engineered *S. cerevisiae* strains SyBE_001188, SyBE_001189, SyBE_001190 and SyBE_001110. Data are represented as mean value of production from three independent fermentations.

In order to produce taxadiene in yeast chassis, an episomal plasmid only containing the *ts* was constructed initially. The *ts* was controlled by a strong constructive promoter *tdh3*, and the plasmid was transformed into W303-1A, obtaining strain SyBE_001188. No taxadiene was detected after 60 h shaking flask fermentation in SD medium. The target compound taxadiene was still undetected when another plasmid containing both *ts* and *ggppssc* was constructed (Strain SyBE_001189). The results indicated that the content of precursor FPP may not be sufficient for production of GGPP leading to the failure of taxadiene production. Efforts to improve the content of FPP were invested with construction of integrated plasmids pRS304-*tdh3p-thmgr-cyct* and pRS403-*tdh3p-erg20-cyct*. The *erg20* cassette was firstly integrated into the genome of strain SyBE_001189 to get strain SyBE_001190. After 60 h shaking flask fermentation, this strain produced 0.22 mg/L taxadiene. Then *thmgr* cassette was integrated into the genome of strain SyBE_001190, yielding strain SyBE_001110 which produced 1.82 mg/L taxadiene (Fig. 2). Consistent with previous report, the results suggested that overexpression of *erg20* and *thmgr* is very important for the terpenes production [13], [14], [22].

### Identification of an optimal GGPPS by computer aided protein modeling

Since conversion of FPP to GGPP was a rate-limiting step for taxadiene synthesis [16], it is envisaged that the taxadiene production of strain SyBE_001110 could benefit from optimization of GGPP production. Therefore, our effort shifted towards the search of more efficient GGPPS to increase taxadiene production. The conversion efficiency of GGPPS is determined primarily by the catalytic capability and fitness with yeast chassis. Ideally, the biochemical properties (Km, kcat etc.) of GGPPS from different species should be taken consideration in the process of selection. However, there are very limited *in vitro* characterization data of GGPPS in literature [25]. Therefore, selection of GGPPSs to improve the taxadiene production poses a great challenge. It generally requires several rounds of random screening to hopefully identify the best isoenzymes which involves significant efforts.

**Figure 3:**
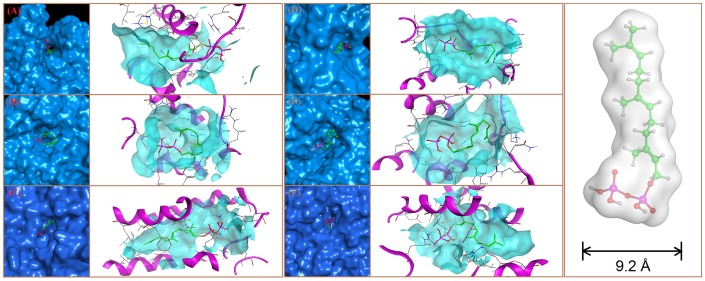
Docking result of FPP with (A) GGPPSbc; (B) GGPPSgb; (C) GGPPSrc; (D) GGPPSeh; (E) GGPPScr; (F) GGPPSsc. In A-F, the left figure represent the whole protein-substrate docking; the right figure is site gain.

Computer aided drug design has been well established for drug discovery to design the inhibitor with higher binding affinity through the study of the interaction between inhibitor and protein target at molecular level. Hypothetically, in selection of isoenzymes that lack of biochemical properties, protein modeling and docking could be utilized to choose the isoenzymes with higher affinity towards substrate. Protein modeling study [28] was performed on six GGPPSs composed of GGPPSsc and another five GGPPSs originated from *T. baccata x T. cuspidate* (GGPPSbc), *G. biloba* (GGPPSgb), *R. catesbeiana* (GGPPSrc), *E. herbicola* (GGPPSeh) and *C. reinhardtii* (GGPPScr) (Fig. 3). The sizes of the pocket of GGPPSs would affect the catalytic rate to a large extent. Specifically, since the width of simulated FPP in vacuum was approximately 9.2∶Å (Fig. 3G), the narrow orifices of GGPPSrc (8.2∶Å) and GGPPSsc (8.6∶Å) were likely to thwart the entry of FPP, which may result in difficulties on transit of the substrate-FPP and the product-GGPP. In contrast, the larger aperture of GGPPSbc (11.4∶Å), GGPPSgb (10.6∶Å), GGPPScr (10.8∶Å) and GGPPSeh (9.6∶Å) were unhindered for the ligand (Fig. 3). As the aperture of the GGPPS is large enough, the ligand FPP could pass freely. Another factor would contribute to the distinctions of catalytic efficiencies among the latter three enzymes, namely the stability of ligand-receptor complex in equilibrium that could be characterized by the binding affinity. The affinity of six GGPPSs with FPP was scored and ranked (Table 2). The enzymes GGPPSbc and GGPPSgb with deep, well-defined pockets usually favor the binding of the substrate than others and show better catalytic capability. Thus, it was predicted from the protein-substrate docking result that the different binding modes of the six GGPPS catalyzing FPP to GGPP might lead to the different conversion efficiency from FPP to GGPP which may result in different taxadiene yield, and GGPPSbc might perform the best. Therefore, GGPPSbc, GGPPSeh and GGPPSsc that predicted to perform the best, medium and worst were selected for further experiment in hope of validating our prediction.

**Table 2:** Data of enzyme-substrate docking.

Enzyme	Binding energy (Kcal/mol)	Instant constant (µM)	Estimated rank
GPPSbc^1^	−8.09	1.17	1
GGPPSgb	−7.17	5.58	2
GGPPSrc	−6.20	28.59	3
GGPPSeh^1^	−5.88	48.80	4
GGPPScr	−5.49	62.37	5
GGPPSsc^1^	−4.90	256.57	6

1 The GGPPSs selected for the construction of artificial cells.

### The Fitness between GGPPS and Chassis

**Figure 4:**
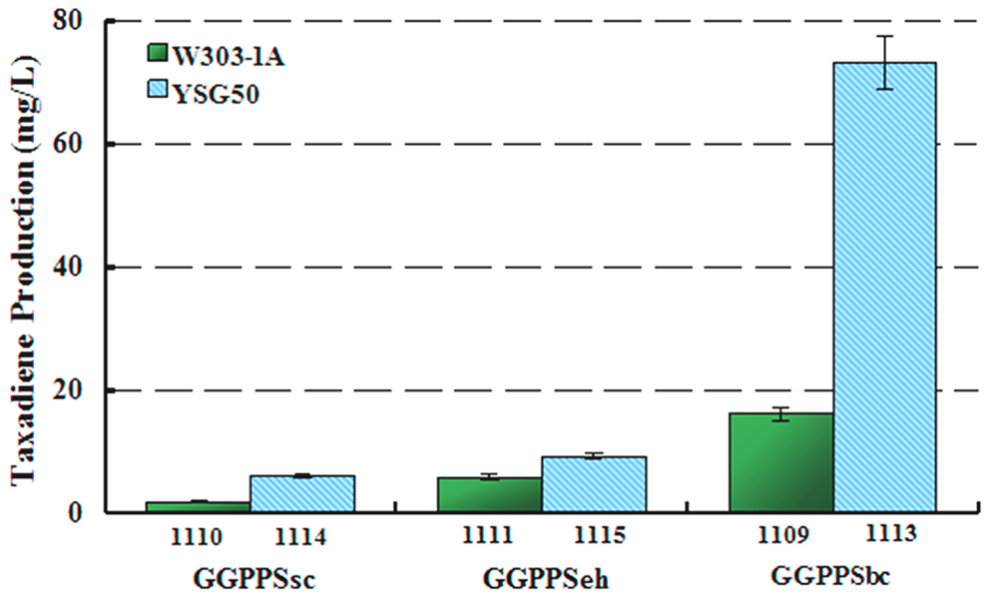
Production of taxadiene by engineered *S. cerevisiae* strains SyBE_001109, SyBE_001110, SyBE_001111, SyBE_001113, SyBE_001114, and SyBE_001115. Data are represented as mean value of production from three independent fermentations.

The adaptation of heterologous enzymes to the yeast chassis is a key point for their high performance. We constructed plasmids which expressed *ts* and *ggppseh* or *ggppsbc*. Strain SyBE_001104 was firstly obtained after introducing the modules *tdh3p-erg20-cyct* and *tdh3p-thmgr-cyct* into chassis W303-1A. Then the plasmids were transformed into strain SyBE_001104 separately to get strain SyBE_001111 (W303-1A with *ggppseh)* and SyBE_001109 (W303-1A with *ggppsbc*). The yield of SyBE_001111 and SyBE_001109 were 1.5 and 7.2 fold over the strain SyBE_001110 (W303-1A with *ggppssc*) (Fig. 4). Based on the results, choosing more capable isoenzymes in the microbial metabolic engineering pathways might result in a significant improvement on terpenoids production.

Strain SyBE_001103 was obtained by inducing the modules *tdh3p-erg20-cyct* and *tdh3p-thmgr-cyct* into YSG50. Transforming episomal plasmids containing *ts* and *ggppssc* or *ggppseh* or *ggppsbc* separately into strain SyBE_001103 yielded strain SyBE_001114, SyBE_001115 and SyBE_001113, respectively. Shaking flask fermentation results showed that there were improvements of taxadiene production with *ggppssc, ggppseh* or *ggppsbc* in YSG50. The yield of strain SyBE_001114 (YSG50 with *ggppssc*), SyBE_001115 (YSG50 with *ggppseh*), SyBE_001113 (YSG50 with *ggppsbc*) were 3.7, 1.6 and 4.3 fold to strain SyBE_001110 (W303-1A with *ggppssc*), SyBE_001111 (W303-1Awith *ggppseh*) and SyBE_001109 (W303-1A with *ggppsbc*) (Fig. 3).

The performances of strains containing different GGPPS in both W303-1A and YSG50 were consistent with the efficiency rank in computational docking. It indicated that computer aided protein modeling guided isoenzyme selection could be a good strategy for design and construction in synthetic biology.

The highest taxadiene production was obtained in strain SyBE_001113, which overexpressed *thmgr* and *erg20* in two integrated plasmids and overexpressed *ggppsbc* and *ts* in a high-copy plasmid. The results described above suggested that W303-1A and YSG50 had different abilities for taxadiene production and YSG50 was more capable for this specific system. The conclusion was further validated by the following metabolomic study that YSG50 was more suitable for taxadiene production.

Another interesting result is that the western blotting experiments performed on yeasts with GGPPSbc, GGPPSeh, and GGPPSsc showed different expression levels with the docking and GGPP production experimental results (Fig. S3a in File S1). In addition, the real time PCR analysis (Fig. S3b in File S1) also showed that the expression level of *ggppssc* was significantly higher than that of *ggppseh* and *ggppsbc*, which further confirmed the western blotting result. The highest expression level of GGPPSsc may lead to the result that the fitness between yeast chassis and GGPPSsc should be better than that between yeast and GGPPSs from GGPPSbc and GGPPSeh. However, the fitness between GGPPSsc and the downstream enzymes taxadiene synthase from *T. brevifolia* was not so satisfied and the yield of taxadiene was worse than others. It was speculated that the catalytic efficiency of GGPPSbc and GGPPSeh were significantly higher than that of GGPPSsc, though the expression level of GGPPSsc is significantly high. On the other hand, as we have discussed above, the sizes of the pocket of GGPPSs and the binding affinity of GGPPSs with FPP could affect the catalytic rate to a large extent. Thus, it could further conclude that the enzyme activity GGPPSs is mainly dependent on the structure of GGPPSs, not on the expression level.

### Metabolomic study for choosing more suitable chassis

The genetic background of host may have dramatic effect with regard to recombinant terpene production [7]. Thus, another yeast YSG50 with different genetic background [34] was chosen for taxadiene production. Metabolomics study was carried out to compare the primary metabolic pathway of chassis YSG50 and W303-1A.

**Figure 5:**
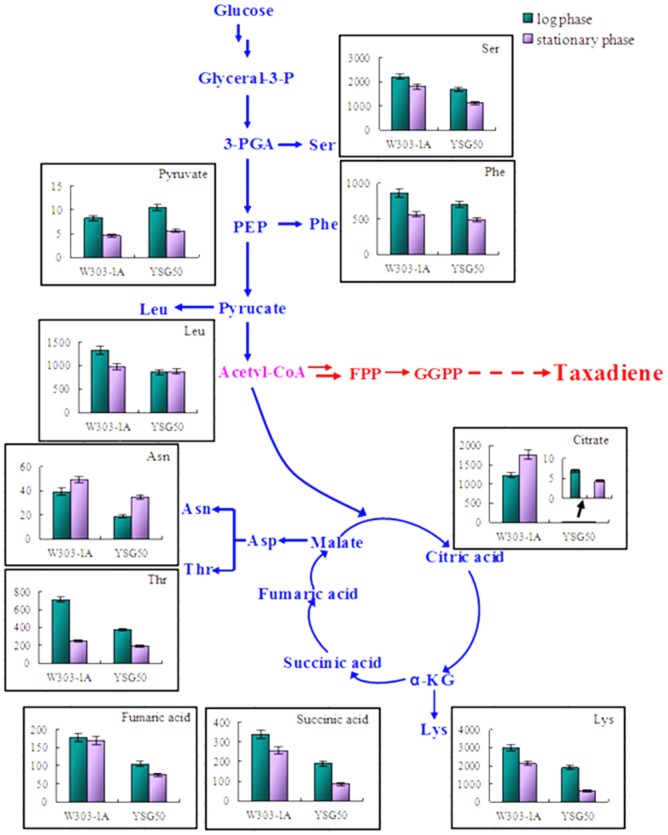
The contents of identified metabolites and amino acids in glycolytic pathway and TCA cycle of W303-1A and YSG50. The y-axis was relative abundance which was calculated by normalization of peak area of each metabolite to internal standard, and each value represented mean value of two independent replicates.

TCA cycle is one of the competitive ways of MVA pathway. The analysis of TCA cycle intermediates may give us some evidence for further constructing the taxadiene producing yeast. Ten metabolites which were related to glycolytic pathway and TCA cycle were identified (Fig. 5). It was found that the contents of pyruvate in glycolytic pathway were almost the same in the two chassis. The contents of three amino acids (Ser, Phe and Leu) which were branched from glycolytic pathway were higher in W303-1A than those in YSG50. Thus, more carbon might flux into the synthesis of acetyl-CoA in YSG50. From the branch point acetyl-CoA, the carbon flux divided into several pathways including TCA cycle and MVA pathway. The contents of three identified metabolites (citrate, fumarate, and succinate) in TCA cycle were all higher in chassis W303-1A. Furthermore, the contents of Thr and Asn whose precursors were synthesized in TCA cycle were also higher in W303-1A. Therefore, less carbon flux divided into TCA cycle in chassis YSG50 which may result in more carbon flux in MVA pathway.

Shalel-Levanon *et al.* [35] found that the expression levels of genes in TCA cycle (e.g. icd, gltA, sucA, sucC, and sdhC) in *E. coli* during microaerobic growth increased as the increase of oxygen concentration, which indicated that the TCA cycle was inhibited by the oxygen availability. Restriction of oxygen could inhibit TCA cycle and increase the flux of MVA. Thus, the effects of dissolved oxygen on TCA cycle of SyBE_001115 were investigated as shown in Fig. S4 in File S1. It could be seen that the total production of taxadiene decreased as the decrease of airflow rate. However, the cell mass (OD_600_) was also affected that the production of per cell increased significantly. It indicated that more carbon flux was split into MVA pathway if the TCA cycle is affected. It was reported that 2-ketoglutarate dehydrogenase is repressed blocking the TCA cycle at 2-oxoglutarate in oxygen limited conditions, and pyruvate can then be metabolized to acetyl-CoA [36]. In our taxadiene producing yeast, more acetyl-CoA under low airflow rate could be converted to taxadiene via MVA pathway.

**Figure 6:**
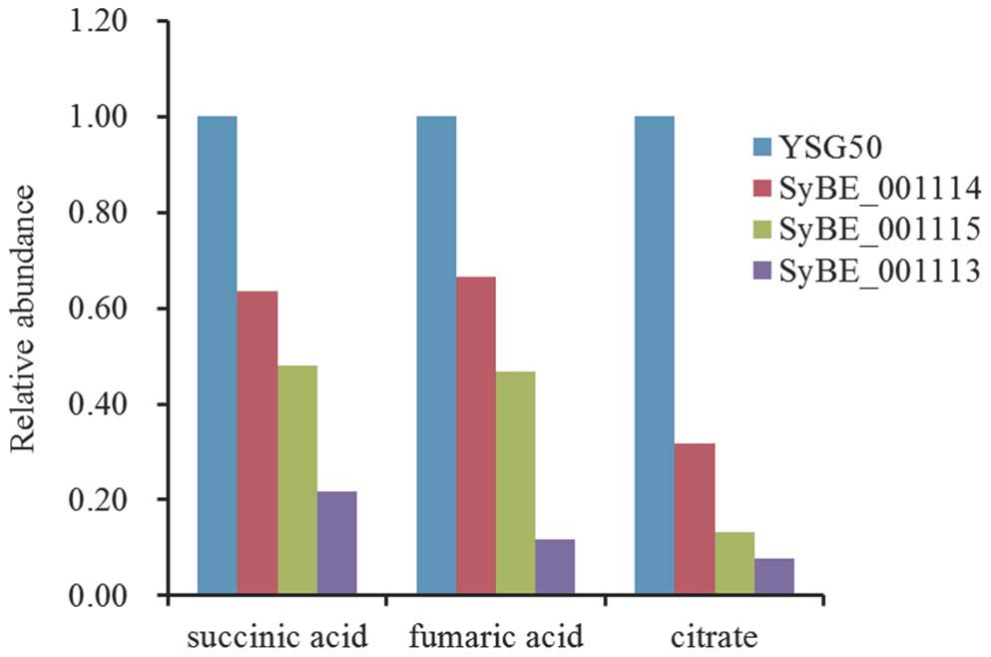
The relative abundance of TCA cycle intermediates (citrate, succinic acid and fumaric acid) in taxadiene producing yeast SyBE_001113, SyBE_001114, and SyBE_001115 compared with those in YSG50 chassis.

Moreover, another metabolomics experiment was performed on three functional yeasts (SyBE_001113, SyBE_001114, and SyBE_001115) constructed for biosynthesis of taxadiene compared with their chassis YSG50. As shown in Fig. 6, the relative abundance of the TCA intermediates (citrate, succinic acid, and fumaric acid) in the taxadiene biosynthetic yeasts were lower than the chassis strain (YSG50). It indicated that the TCA cycle was significantly affected by the biosynthesis of taxadiene. Thus, it could further support our hypothesis that more carbon flux in MVA pathway was benefit for the production of taxadiene.

### Conclusions

A stable genome-transformed *S. cerevisiae* strain with taxadiene titer of 72.8 mg/L was obtained by studying of the fitness of yeast chassis with different GGPPSs guided by computer aided protein modeling and metabolomic study. The yield rank of the engineered strains was consistent with the efficiency rank of GGPPS in computational docking, which demonstrated that the enzyme-substrate docking has the potential to be a useful tool for identification of efficient enzymes and rational design of synthetic strains with better performance. In addition to GGPPS, the computer aided protein modeling guided selection strategy could be used to assist predicting more efficient isoenzymes in many other enzymes on this biosynthesis pathway (such as HMGR). The further improvement of the overall production yield of taxadiene in yeast chassis would eventually provide a yeast taxadiene production option that are more suitable for further construction for biosynthesis steps and industrial production than *E. coli*.

## Supporting Information

File S1 **Contains the following files.** Figure S1, (a) ^1^H NMR spectra of taxadiene. 400 MHz, CDCl3: 5.3(t, J = 4.8 Hz, 1H), 2.04–1.90 (m, 14H), 1.61 (s, 6H), 1.53 (s, 9H). (b) ^1^C NMR spectra of taxadiene. 100 MHz, CDCl3: 124.40, 124.19, 123.79, 123.29, 59.40, 39.73, 39.70, 39.57, 26.77, 26.63, 26.33, 25.70, 17.69, 16.29, 16.02. Figure S2, Production of taxadiene by engineered *S. cerevisiae*. This strain was cultivated in SD medium with 2% glucose for 66 hour. A: GC-MS analysis of n-Hexane extracts. B: Mass spectra of taxadiene. Figure S3, (a) Western blotting of different GGPPS during exponential growth of yeast. (b) Relative expression of different *ggpps* analyzed by real-time PCR. Figure S4, Cell mass (OD_600_) and taxadiene production of SyBE_001115 under different airflow rates. Table S1, Primers used in constructing procedure. Table S2, Homology modeling templates information.
(DOCX)
