# Supplementary material for: Biosynthesis of Taxadiene in Saccharomyces cerevisiae : Selection of Geranylgeranyl Diphosphate Synthase Directed by a Computer-Aided Docking Strategy
Source: PLoS One. 2014 Oct 8;9(10):e109348. doi: 10.1371/journal.pone.0109348 (PMC4190181; doi:10.1371/journal.pone.0109348)
Supplement: File S1 — Contains the following files. Figure S1, (a) 1H NMR spectra of taxadiene. 400 MHz, CDCl3: 5.3(t, J = 4.8 Hz, 1H), 2.04–1.90 (m, 14H), 1.61 (s, 6H), 1.53 (s, 9H). (b) 1C NMR spectra of taxadiene. 100 MHz, CDCl3: 124.40, 124.19, 123.79, 123.29, 59.40, 39.73, 39.70, 39.57, 26.77, 26.63, 26.33, 25.70, 17.69, 16.29, 16.02. Figure S2, Production of taxadiene by engineered S. cerevisiae. This strain was cultivated in SD medium with 2% glucose for 66 hour. A: GC-MS analysis of n-Hexane extracts. B: Mass spectra of taxadiene. Figure S3, (a) Western blotting of different GGPPS during exponential growth of yeast. (b) Relative expression of different ggpps analyzed by real-time PCR. Figure S4, Cell mass (OD600) and taxadiene production of SyBE_001115 under different airflow rates. Table S1, Primers used in constructing procedure. Table S2, Homology modeling templates information. (DOCX) [file pone.0109348.s001.docx]

**Supporting Information:**

**Table S1 Primers used in constructing procedure**

| **Primer No.** | **Name** | **Sequence** |
| --- | --- | --- |
| 1 | 304tH -F | 5’-CGGAATTCATGGTTTTAACCAATAAAACA-3’ |
| 2 | 304tH-R | 5’-GTTCTGCAGTTAGGATTTAATGCAGGTGA-3’ |
| 3 | 304T3-F | 5’-CTTGGGCCCATAAAAAACACGCTTTTTCAG-3’ |
| 4 | 304T3-R | 5’-CGGAATTCTTGTTTGTTTATGTGTGTTTAT-3’ |
| 5 | 304Ct-F | 5’-GTTCTGCAGATTAGTTATGTCACGCTTACATTCA-3’ |
| 6 | 304Ct-R | 5’-CGGGATCCAGCTTGCAAATTAAAGCCTT-3’ |
| 7 | 403E20-F | 5’-CGGGATCCATGGCTTCAGAAAAAGAAATTA-3’ |
| 8 | 403E20-R | 5’-CGGAATTCCTATTTGCTTCTCTTGTAAAC-3’ |
| 9 | 403T3-F | 5’-GCTCTAGAATAAAAAACACGCTTTTTCAG-3’ |
| 10 | 403T3-R | 5’-CGGGATCCTTGTTTGTTTATGTGTGTTTAT-3’ |
| 11 | 403Ct-F | 5’-CGGAATTCATTAGTTATGTCACGCTTACATTCA-3’ |
| 12 | 403Ct-R | 5’-CGGGGCCCAGCTTGCAAATTAAAGCCTT-3’ |
| 13 | BTS-F | 5’-ATGGAGGCCAAGATAGATGA-3’ |
| 14 | BTS-R | 5’-TCACAATTCGGATAAGTGGT-3’ |
| 15 | 425T3-F | 5’-TCGAGCTCATAAAAAACACGCTTTTTCAG-3 |
| 16 | 425T3-R | 5’-TTGTTTGTTTATGTGTGTTTATTTGAAAGATAATTGTGCCAT-3’ |
| 17 | TS-F | 5’-ATGGCACAATTATCTTTCAATGCC-3’ |
| 18 | TS-R | 5’-TTCAATTCAATTCAATTTATTAAACTTGGATAGGGTC-3’ |
| 19 | 425Pt-F | 5’-TAAATTGAATTGAATTGAAAT-3’ |
| 20 | 425Pt-R | 5’-AACGAACGCAGAATTTTCG-3’ |
| 21 | btP1-F | 5’-CCAAGCTTTATTTTAGATTCCTGACTTCAAC-3’ |
| 22 | btP1-R | 5’-TCATCTATCTTGGCCTCCATTGTTTTATATTTGTTGTAAA-3’ |
| 23 | OEBTS-F | 5’-ATGGAGGCCAAGATAGATGAG-3’ |
| 24 | OEBTS-R | 5’-TCACAATTCGGATAAGTGTCACAATTCGGATAAGTG-3’ |
| 25 | btCt-F | 5’-ATTAGTTATGTCACGCTTAC-3’ |
| 26 | btCt-R | 5’-TTGGGCCCAGCTTGCAAATTAAAGCCTT-3’ |
| 27 | crP1-F | 5’-CCAAGCTTTATTTTAGATTCCTGACTTCAAC-3’ |
| 28 | crP1-R | 5’-CCTTAGAACCAGAAACCATTGTTTTATATTTGTTGTAAA-3’ |
| 29 | crtE-F | 5’-ATGGTTTCTGGTTCTAAGGCTG-3’ |
| 30 | crtE-R | 5’-TCACAATTCGGATAAGTGTTAAGCTATCTTCATGACA-3’ |
| 31 | crCt-F | 5’-ATTAGTTATGTCACGCTTAC-3’ |
| 32 | crCt-R | 5’-CTTGGGCCCAGCTTGCAAATTAAAGCCTT-3’ |
| 33 | gpP1-F | 5’-CCAAGCTTTATTTTAGATTCCTGACTTCAAC-3’ |
| 34 | pgP1-R | 5’-GCCATTGCGGTATAAGCCATTGTTTTATATTTGTTGTAAA-3’ |
| 35 | GPbc-F | 5’-ATGGCTTATACCGCAATGGCAGC-3’ |
| 36 | GPbc-R | 5’-TCACAATTCGGATAAGTGTTAGTTTTGCCTGAAAGCG-3’ |
| 37 | gpCt-F | 5’-ATTAGTTATGTCACGCTTAC-3’ |
| 38 | gpCt-R | 5’-CTTGGGCCCAGCTTGCAAATTAAAGCCTT-3’ |

**Table S2. Homology modeling templates information**

| Enzyme | Template PDB code | Total score | Query coverage | E-value | Identity |
| --- | --- | --- | --- | --- | --- |
| GGPPSbc | 2J1P | 381 | 73% | 1e^-130^ | 65% |
| GGPPSgb | 3KRA | 421 | 75% | 3e^-146^ | 69% |
| GGPPSrc | 2E90 | 541 | 98% | 0 | 86% |
| GGPPSeh | 3KRA | 124 | 65% | 7e^-22^ | 42% |
| GGPPScr | 3KRA | 400 | 84% | 1e^-138^ | 65% |
| GGPPSsc* | 2DH4 | 679 | 100% | 0 | 100% |

*The X-ray crystal structure of GGPPSsc has already been resolved and deposited in Protein Data Bank (code: 2DH4)


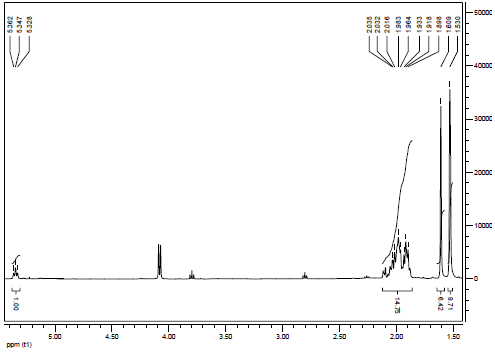


(a)

*
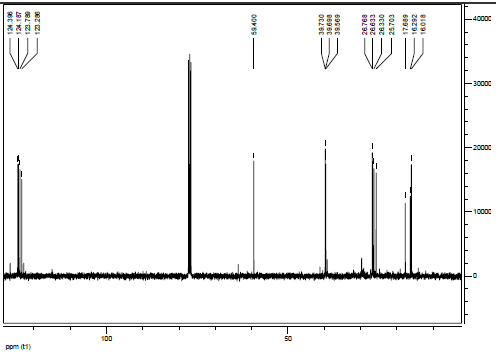
*

(b)

**Fig. S1** (a) **^1^**H NMR spectra of taxadiene. 400MHz, CDCl3: 5.3(t,J=4.8 Hz,1H), 2.04-1.90 (m,14H), 1.61 (s,6H), 1.53 (s,9H); (b) **^1^**C NMR spectra of taxadiene. 100MHz, CDCl3: 124.40, 124.19, 123.79, 123.29, 59.40, 39.73, 39.70, 39.57, 26.77, 26.63, 26.33, 25.70, 17.69, 16.29, 16.02.


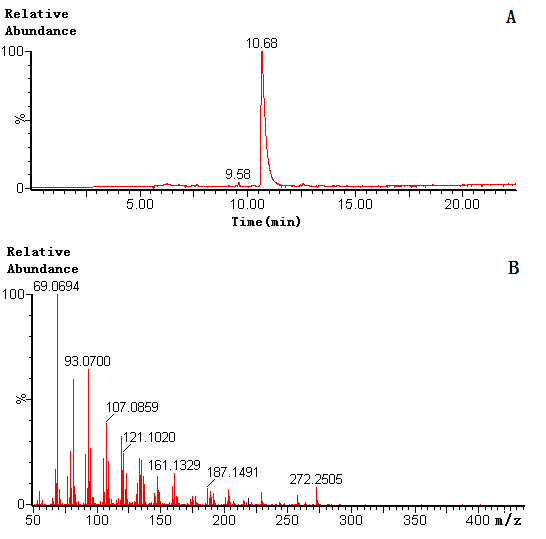


**Fig. S2** Production of taxadiene by engineered *S. cerevisiae*. This strain was cultivated in SD medium with 2% glucose for 66hour. A: GC-MS analysis of n-Hexane extracts. B: Mass spectra of taxadiene.


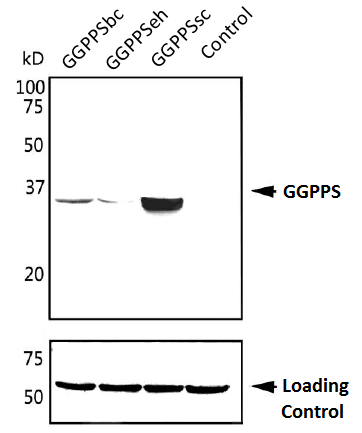


(a)


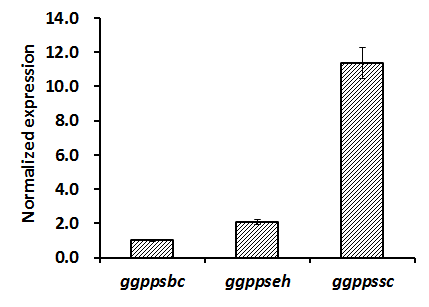


(b)

**Fig. S3** (a) Western blotting of different GGPPS during exponential growth of yeast. (b) Relative expression of different *ggpps* analyzed by real-time PCR.


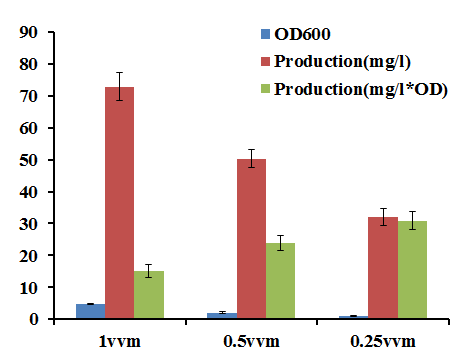


Fig. S4 Cell mass (OD_600_) and taxadiene production of SyBE_001115 under different airflow rates.
